# The effect of follow-up blood cultures on mortality and antibiotic use in gram-negative bloodstream infections

**DOI:** 10.1186/s12879-023-08500-9

**Published:** 2023-08-29

**Authors:** Mehmet Yildiz, Hamid Habibi, Fatma Betul Altin, Seref Kerem Corbacioglu, Hasan Selcuk Ozger

**Affiliations:** 1 Infectious Disease and Clinical Microbiology, Gazi University School of Medicine, Ankara, Turkey; 2 Emergency Departments, Atatürk Sanatorium Training and Research Hospital, Ankara, Turkey

**Keywords:** Gram-negative bloodstream infections, Follow-up blood cultures, Persistent infections, Mortality, Antibiotic consumption

## Abstract

**Background:**

Gram-negative bloodstream infections (GN-BSIs) are a significant clinical challenge. The utility of follow-up blood cultures (FUBCs) in GN-BSIs and their impact on mortality and antibiotic consumption are areas of debate. This study aimed to evaluate the effect of FUBCs on mortality and antibiotic consumption in patients with GN-BSIs.

**Methods:**

This single-center, retrospective study was conducted in aged > 18 years of patients with GN-BSIs. FUBC was defined as a blood culture performed 2–7 days after the first blood culture. Patients were grouped as FUBC and no FUBC and compared. A 1:1 match analysis was performed between the groups according to the SOFA score. The matched subgroup was compared for mortality risk factors with logistic regression models. The two groups were compared for the duration of effective antibiotic therapy and total antibiotic consumption (days of therapy per 1000 patient days (DOT/1000 PD)).

**Results:**

FUBC was performed in 564 (69.4%) of 812 patients. Persistent, positive and negative FUBC rates were 7.9%, 14%, and 78%, respectively. The frequency of persistent GN-BSI in patients with appropriate antibiotic therapy was 3.9%. SOFA score (OR:1.33; 95% CI, 1.23–1.44), Charlson comorbidity index score (OR:1.18; 95% CI, 1.08–1.28), hospital-acquired infections (OR:1.93; 95% CI, 1.08–3.46) and carbapenem-resistant GN-BSI (OR: 2.92; 95% CI, 1.72–4.96) were independent risk factors for mortality. No relationship was found between FUBC and mortality (p > 0.05). Duration of effective antibiotic therapy (10(4–16) vs. 15(9–20), p < 0.001) and DOT/1000 PD (1609 (1000–2178) vs. 2000 (1294–2769), p < 0.001) were longer in the FUBC group.

**Conclusion:**

Routine FUBC should not be recommended because of the low prevalence of persistent infections in patients under appropriate antibiotic therapy and FUBC increases antibiotic consumption.

## Background

Follow-up blood cultures (FUBCs) are frequently used in the management of gram-negative bloodstream infections (GN-BSIs). Unlike *S. au*reus and *Candida* spp. related BSIs, there is no consensus for FUBCs in GN-BSI [1, 2]. Due to the lack of defined standards, the frequency of FUBC for GN-BSI ranges from 18 to 86% in published articles [3–15].

The most frequently evaluated outcome associated with FUBC is mortality. Early detection of persistent and breakthrough infections with routine FUBC in GN-BSIs can reduce mortality through appropriate antimicrobial therapy and early source control. However, this hypothesis has still not been confirmed due to the high risk of bias in published publications [16, 17]. Early mortality before FUBC leads to the misleading association of FUBC with low mortality in these articles. Articles report controversial results for the relationship between mortality and FUBC. However, the common result of many studies is that FUBC prolongs antibiotic exposure [16]. This comparison includes only primary effective antibiotic treatments. It does not include sequential treatments (persistent, breakthrough, contamination, etc.) affected by the FUBC result.

This study aims to evaluate the relationship between FUBC and mortality by reducing confounding factors and determining the effects of FUBC on total antibiotic consumption in the post-culture period.

## Methods

### Study design

This is a single-center, retrospective, and descriptive study between January 2019 and December 2022. The study was approved by the Gazi University Faculty of Medicine Clinical Research and Ethics Committee (approval date November 29, 2021; approval no. 131).

Study population and setting: Patients with gram-negative bacteria in their blood cultures were obtained from the electronic database of the central microbiology laboratory. Patients aged > 18 years who were hospitalized in the medical and surgical services or intensive care units were included in the study. The first GN-BSI episode was included for each patient. Polymicrobial BSIs, and patients who died or were discharged within the first 72 h after index culture were excluded.

### Definitions

The first positive blood culture with GN-bacteria was defined as the index culture, and the blood culture (BC) 2–7 days after the index BC was defined as FUBC [15]. In our center, no diagnostic algorithm was used for the decision of FUBC, FUBCs were taken with the individual decisions of the clinician.

Detection of the same bacteria in index BC and FUBC was defined as persistent BSI. Detection of different bacteria in FUBC was defined as positive BSI and grouped as contaminants and non-contaminants. Bacteria that are commensal skin flora elements (i.e., coagulase-negative staphylococci, *corynebacterium* spp., *Bacillus* spp.) were considered contaminants except for their growth in 2 or more sets of blood culture or the presence of sepsis [18].

BSIs ≥ 48 h after hospital admission were defined as hospital-acquired, others as community-acquired BSI [19]. BSIs were considered secondary BSIs in the presence of focal infection from which the same organism was isolated. Secondary BSIs sources were assessed according to CDC criteria and classified as skin/soft tissue, gastrointestinal, genitourinary, endovascular, and respiratory-lung-related BSI [18]. Resistance to at least one antibiotic from three or more antibiotic categories was defined as multi-drug resistance (MDR), resistance to all antibiotics except polymyxin and/or tigecycline was defined as extreme drug-resistant (XDR), and resistance to all antibiotics was defined as pan-drug resistant (PDR) [20]. Appropriate empirical antibiotic therapy was defined as the parenteral use of an appropriate dose of an in vitro effective antibiotic within the first 24 h after the index blood culture. The duration of the appropriate antibiotic was accepted as the duration of the effective antibiotic. Total antibiotic consumption was calculated as days of therapy per 1000 patient days (DOT/1000 PD) over antibiotics used in index BSIs and antibiotics used within 30 days after index culture [21].

Microbiological identification: Blood cultures at our hospital are incubated using Bactec FX Automated Blood Culture System (Becton Dickinson, Franklin Lakes, NJ, USA). All positive blood cultures are examined by Gram stain, inoculated on 5% sheep blood agar, Eosin Methylene blue (EMB) Agar, and incubated at 35–38 °C for 18–24 h. Microorganism identifications are made with MALDI-TOF MS (Bruker Daltonics, Bremen, Germany) and phenotypic susceptibility tests are made with VITEK 2 (bioMérieux, Marcy l’Étoile, France). Antibiotic susceptibility results are reported according to the European Committee for Antimicrobial Susceptibility Testing (EUCAST) clinical breakpoints.

### Study protocol

Clinical variables and outcomes of patients who met the eligibility criteria were obtained from the electronic medical records. These clinical variables and outcomes included age, sex, comorbidities, invasive devices (central venous catheter, cardiac device, and prosthetic device), site and sources of infection, microorganisms, and resistance profiles, FUBC performed and results (persistent, positive, and negative), antibiotic therapies, DOT/1000 PD and 30-day mortality. The Charlson comorbidity index (CCI), sequential organ failure (SOFA) score, and systemic inflammatory response syndrome (SIRS), scores of the patients were calculated and recorded by the researchers using hospital electronic records. To determine the factors for FUBC, patients were grouped as FUBC and no FUBC and compared. To reduce confounding factors that may affect mortality between groups, a matched subgroup was formed by performing 1: 1 match analysis between the groups with and without follow-up blood culture according to the SOFA score. The matched subgroup was compared for mortality risk factors and antibiotic consumption (duration of effective antibiotic therapy and DOT/1000 PD). To determine the risk factors associated with persistent BSI, patients who performed FUBC were grouped as persistent and non-persistent and compared.

### Outcomes

The primary outcome of the study was to assess the effect of FUBCs on 30-day mortality. The secondary outcome of the study was to determine the effect of FUBC on antibiotic use.

### Statistical analysis

All data were analyzed using SPSS v25.0 for Mac OS X (SPSS Inc., Chicago, IL, USA). The normality of the data distribution was determined by the Shapiro–Wilk test, histograms, and Q-Q plots. The categorical variables of the patients were expressed as numbers and percentages and were analyzed using a chi-square test. Continued variables were presented as the mean standard deviation (SD) or median values and interquartile range (IQR) of 25–75%. Nonparametric values were analyzed using the Mann–Whitney *U* test, and parametric values were analyzed using the student’s *t*-test. To control the risk of bias that may arise from the possible inverse correlation between FUBC and survivors, a 1:1 match analysis was performed between the groups with and without FUBC according to the SOFA score. To determine the predictive value of the variables in the matched group, those with a p-value of < 0.05 in the univariate analysis were entered into a multivariate regression model using the block-wise entry method-hierarchical. Correlations among these variables were analyzed using Spearman’s test. In each pair, the variable that detected a high correlation (rho > 0.75) with the other variable was excluded from the regression model. To assess the model’s goodness of fit, the Hosmer–Lemeshow test was performed. The 95% confidence intervals (95% CIs) were calculated whenever appropriate, and a two-tailed *p*-value of < 0.05 was considered statistically significant. The power analysis was calculated according to 30-day mortality with G Power (version 3.1.9.6).

## Results

**Fig. 1:**
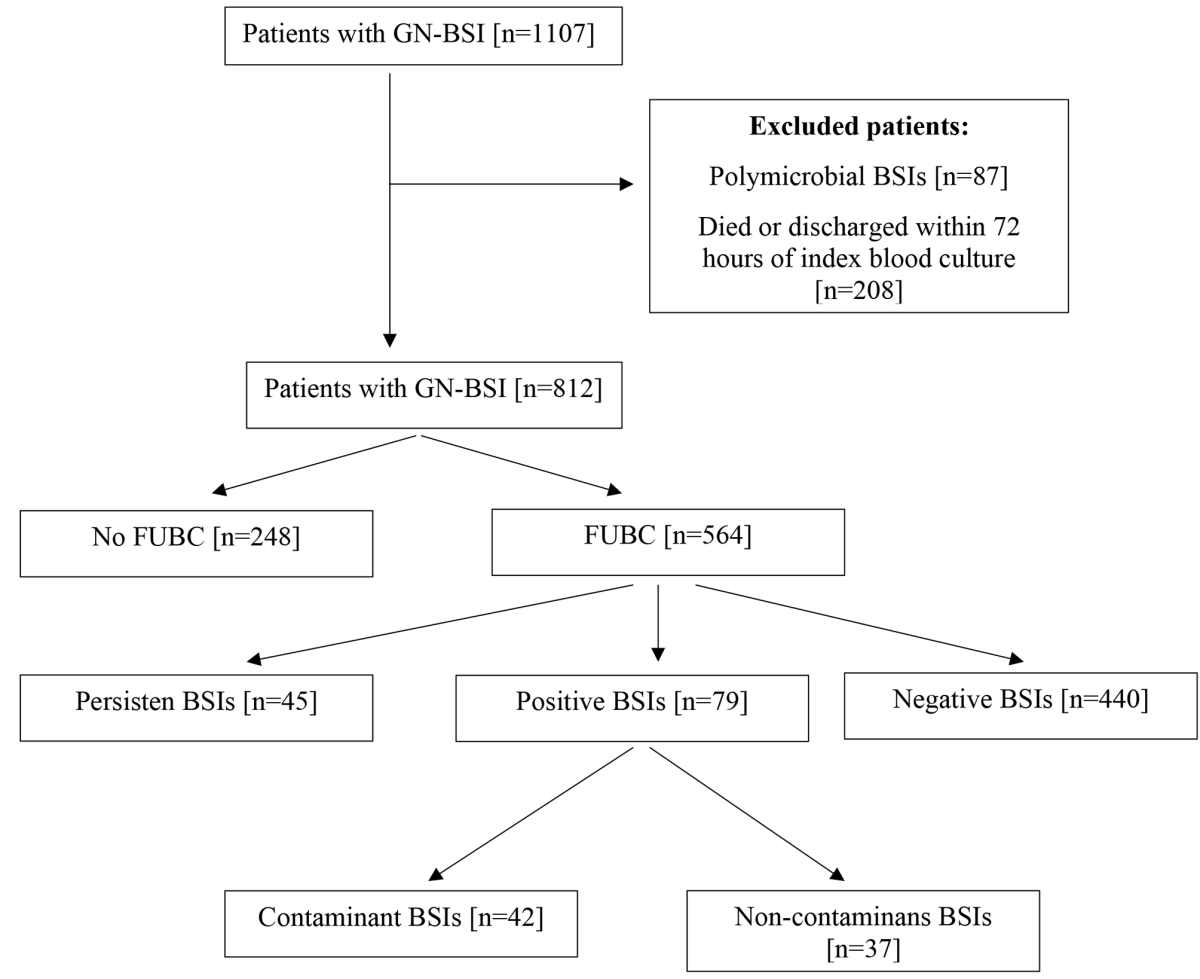
Flowchart of study **Abbreviations:** GN-BSI: gram-negative bloodstream infection, FUBC: follow-up blood culture

812 patients were included in the study. 564 (69.4%) patients had FUBC. The average time between the FUBC and the index culture was 3(4–5) days. FUBC was persistent, positive, and negative FUBC rates were 7.9% (n = 45), 14% (n = 79), and 78% (n = 440), respectively. (Fig. 1) The frequency of persistent GN-BSI in patients with appropriate antibiotic therapy was 3.9% (n = 22). The most common agents in persistent GN-BSI were *Enterobacterales* 44.4% (n = 20), *S. maltophlia* 17.8% (n = 8), and *Acinetobacter baumannii* 11.1% (n = 5), respectively. The most common agents in positive BSI were *Enterococcus spp.* 15.5% (n = 12), *Enterobacterales* 12.7% (n = 10) and Candida spp. 7.6% (n = 6), respectively (Table 1).

**Table 1:** BSI pathogens distribution, n (%)

**BSI pathogens**	812 (100)
Enterobacterales	532 (65.5)
*Escherichia coli*	247 (30.4)
*Klebsiella* spp.	197 (24.3)
*Enterobacter* spp.	48 (5.9)
*Serratia* spp.	12 (1.5)
*Proteus* spp.	12 (1.5)
*Morganella* spp.	7 (0.9)
*Salmonella* spp.	6 (0.7)
*Citrobacter* spp.	3 (0.4)
*Acinetobacter baumannii*	82 (10.1)
*Stenotrophomonas maltophlia*	62 (7.6)
*Pseudomonas aureginosa*	61 (7.5)
*Ralstonia insidiosa*	21 (2.6)
*Burkholderia cepacia*	14 (1.7)
*Brucella* spp.	6 (0.7)
Others*	34 (4.2)
**Persistent BSI pathogens**	45 (100)
Enterobacterales	
*Klebsiella* spp	13 (28.8)
*Escherichia coli*	3 (6.7)
*Salmonella* spp.	2 (4.5)
*Morganella* spp.	1 (2.2)
*Citrobacter* spp	1 (2.2)
*S. maltophlia*	8 (17.8)
*A. baumannii*	5 (11.1)
*Brucella* spp.	4 (8.9)
*Ralstonia insidiosa*	3 (6.7)
*P. aureginosa*	2 (4.4)
*Burkholderia cepacia*	2 (4.4)
*Ochrobactrum anthropi*	1 (2.2)
**Positive BSI pathogens**	79(100)
*Enterococcus* spp.	12 (15.2)
Enterobacterales	10 (12.7)
*Klebsiella* spp.	6 (7.6)
*Escherichia coli*	2 (2.5)
*Enterobacter* spp.	1 (1.3)
*Proteus* spp	1 (1.3)
Candida spp.	6 (7.6)
*S. maltophlia*	3 (3.8)
*A.baumannii*	1 (1.3)
*P. aureginosa*	1 (1.3)
*Ochrobactrum anthropi*	1 (1.3)
Coagulase-negative staphylococci**	40 (50.6)
*Corynebacterium* spp.**	2 (4.8)
Polymicrobial pathogens	3 (3.8)

Abbreviation: BSI: bloodstream infection

* Ochrobactrum anthropi, Achromobacter spp, Aeromonas spp, Delftia acidovorans, Moraxella catarrhalis, Raoultella spp., Pantoea spp., Shewanella spp., Sphingomonas spp.

** Considered as a contaminant

Risk factors for persistent BSI are compared and presented in Table 2. High SOFA score, a central venous catheter (CVC), hospital-acquired infection, non-fermenter GN-BSI, carbapenem resistance GN-BSI, and inappropriate empirical antibiotic therapy were found to be risk factors associated with persistent BSI. (p < 0.05) (Table 2).

**Table 2:** Comparison of characteristics of Persistent BSIs and No persistent BSIs

	Persistent (n = 45)	No persistent BSI (n = 519)	P value
Age, median (IQR)	60 (46–74)	64 (55–76)	0.130
Male sex, n (%)	25 (55.6)	294 (56.69	0.887
CCI (n), median (IQR)	4 (2–6)	5 (3–7)	0.152
SOFA, median (IQR)	4 (1–8)	4 (2–6)	0.014
SIRS, median (IQR)	2 (1–2)	2 (1–3)	0.493
ICU, n (%)	22 (48.9)	207 (39.9)	0.238
Central venous catheter, n (%)	29 (64.4)	220 (42.4)	0.004
Cardiac device, n (%)	6 (13.3)	40 (7.7)	0.186
Prosthetic device, n (%)	1 (2.2)	22 (4.2)	0.512
Hospital-acquired BSI, n (%)	33 (73.3)	297 (57.2)	0.035
Primary BSI, n (%)	16 (35.6)	228 (43.9)	0.277
Secondary BSI, n (%)	29 (64.4)	291 (56.1)	
Fermenter GN*, n (%)	20 (44.4)	362 (69.7)	0.001
Non-fermenter GN**, n (%)	25 (55.6)	157 (30.3)	
CR-GN, n (%)	16 (48.5)	114 (23.7)	0.002
MDR, n (%)	35 (77.8)	356 (68.6)	0.200
XDR, n (%)	10 (22.2)	66 (12.7)	0.073
PDR, n (%)	1 (2.2)	5 (1.0)	0.430
Appropriate empirical antibiotic therapy, n (%)	10 (22.2)	307 (59.2)	< 0.001

Abbreviations: FUBC: follow-up blood culture, ICU: intensive care unit, IQR: interquartile range, SOFA score: the sequential organ failure assessment score, SIRS: systemic inflammatory response syndrome, BSI: bloodstream infection, CR-GN: carbapenem-resistant gram-negative, MDR: multi-drug resistant, XDR: extensively drug-resistant, PDR: pan-drug resistant

* Escherichia coli (n=173), Klebsiella spp. (n=142), Enterobacter spp. (n=35), Serratia spp. (n=8), Proteus spp. (n=7), Salmonella spp. (n=5), Citrobacter spp. (n=2),Morganella spp. (n=5), Raoultella ornithinolytica (n=2), Aeromonas spp. (n=3)

** Achromobacter spp. (n=5), Acinetobacter baumannii (n=50), Brucella spp. (n=5), Burkholderia cepacia (n=8), Delftia acidovorans (n=5), Moraxella catarrhalis (n=2), Ochrobactrum anthropi (n=1), Pseudomonas aureginosa (n=37), Pantoea agglomerans (n=2), Ralstonia insidiosa (n=19), Shewanella spp. (n=3), Sphingomonas spp. (n=1), Stenotrophomonas maltophlia (n=44)

Factors for FUBC were compared and presented in Table 3. SOFA score, cardiac device, and ICU support were found to be factors associated with FUBC (p < 0.05).

**Table 3:** Comparison of characteristics of FUBC and No-FUBC

	Overall cohort			Propensity matching cohort		
	FUBC ( n = 564)	No FUBC (n = 248 )	P value	FUBC ( n = 236)	No FUBC (n = 236 )	P Value
Age, median (IQR)	64 (54–75)	65 (55–77)	0.395	64 (51–75)	65 (53.5–76)	0.385
Male sex, n (%)	319 (56.6)	139 (56)	0.892	130 (55.1)	131 (55.5)	0.926
Comorbidities, n (%)						
Diabetes mellitus	163 (28.9)	74 (29.8)	0.787	54 (22.9)	71 (30.1)	0.076
Hypertension	234 (41.5)	96 (38.7)	0.458	96 (40.7)	94 (39.8)	0.851
Chronic renal Failure	86 (15.2)	38 (15.3)	0.978	33 (14.0)	35 (14.8)	0.793
Coronary artery disease	119 (21.1)	47 (19)	0.485	50 (21.2)	44 (18.6)	0.489
Cardiac failure	57 (10.)	24 (9.7)	0.851	19 (8.1)	22 (9.3	0.624
Cerebrovascular disease	66 (11.7)	30 (12.1)	0.873	31 (13.1)	29 (12.3)	0.782
Chronic pulmonary disease	58 (10.3)	26 (10.5)	0.931	24 (10.2)	26 (11.0)	0.765
Malignancy	264 (46.8)	120 (48.4)	0.678	128 (54.2)	116 (49.2)	0.269
CCI(n), median (IQR)	5 (3–7)	5 (3–7)	0.250	5 (3–7)	5 (3–7)	0.481
Clinical severity at index blood culture time, median(IQR)						
SOFA	4 (2–6)	4 (2–7)	0.011	4 (2–7)	4 (2–7)	0.994
SIRS	2 (1–3)	2 (1–3)	0.161	2 (1–3)	2 (1–3)	0.509
Admission ward, n (%)						
ICU	229 (40.6)	128 (51.6)	0.004	111(47.0)	116 (49.2)	0.645
Non-ICU	335 (73.6)	120 (35.9)		125 (53.0)	120 (50.8)	
Invasive device, n (%)						
Central venous catheter	249 (44.1)	113 (45.)	0.709	168 (71.2)	101 (42.8)	< 0.001
Cardiac device	46 (8.2)	8 (3.2)	0.009	13 (5.5)	7 (3.0)	0.170
Prosthetic device	23 (4.1)	8 (3.2)	0.559	12 (5.1)	8 (3.4)	0.361
Site of BSI acquisition, n (%)						
Community-acquired	234 (41.4)	93 (37.5)	0.286	63 (26.7)	91 (38.6)	0.006
Hospital-acquired	330 (58.5)	155 (62.5)		173 (73.3)	145 (61.4)	
Source of BSI, n (%)						
Primary BSI	244 (43.2)	104 (41.9)	0.725	100 (42.4)	100 (42.4)	1.00
Secondary BSI	320 (56.8)	144 (58.1)		136 (57.6)	136 (57.6)	
Skin and soft tissue infection	16 (2.8)	6 (2.4)		5 (2.1)	6 (2.5)	
Gastrointestinal tract infection	41(7.3)	20 (8.1)		9 (3.8)	20 (8.5)	
Genitourinary tract infection	87 (15.4)	34 (13.7)		16(6.8)	33 (14.0)	
Respiratory tract infection	37 (6.6)	29 (11.7)		17 (7.2)	27 (11.4)	
Central venous catheter infection	135 (23.9)	55 (22.2)		87 (36.9)	50 (21.2)	
Other infection	4 (0.7)	0 (0)		2 (0.8)	0 (0)	
Microorganisms in BSI, n (%)						
Enterobacterales	377 (66.8)	155 (62.5)	0.230	143 (60.6)	150 (63.6)	0.507
*Escherichia coli*	173 (30.7)	74 (29.8)		59 (25.0)	72 (30.6)	
*Klebsiella* spp.	142 (25.1)	55 (22.2)		62 (26.3)	53 (22.5)	
*Enterobacter* spp.	35 (6.2)	13 (5.2)		13 (5.5)	12 (5.1)	
*Serratia* spp.	8 (1.4)	4 (1.6)		3 (1.3)	4 (1.7)	
*Proteus* spp.	7 (1.2)	5 (2.09)		2 (0.8)	5 (2.1)	
*Morganella* spp.	5 (0.9)	2 (0.8)		3 (1.3)	2 (0.8)	
*Salmonella* spp.	5 (0.9)	1 (0.4)		1 (0.4)	1 (0.4)	
*Citrobacter* spp.	2 (0.4	1 (0.4)		0 (0.0)	1 (0.4)	
*P. aureginosa*	37 (6.6)	24 (9.7)	0.121	16 (6.8)	24 (10.2)	0.186
*A.baumannii*	50 (8.9)	32 ( 12.9)	0.079	34 (14.4)	28 (11.9)	0.414
*S. maltophlia*	44 (7.8)	18 (7.3)	0.788	20 (8.5)	216 (6.8)	0.488
Others*	56 (9.9)	19 (7.7)	0.304	23 (9.7)	18 (7.6)	0.414
CR-GN	130 (25.3)	72 (31.4)	0.082	74 (34.7)	65 (29.7)	0.260
MDR	173 (30.7)	83 (32.4)	0.430	73 (47.4)	81 (52.6)	0.432
XDR	76 (13.5)	38 (15.3)	0.485	42 (17.8)	34 (14.4)	0.316
PDR	6 (1.1)	7 (2.8)	0.066	3 (1.3)	6 (2.5)	0.313
Follow-up duration (days), median (IQR)	17 (11–27)	12 (6–24)	< 0.001	17 (11–30)	12 (7–24)	< 0.001
Follow-up duration days in patients without mortality, median (IQR)	18 (13–30)	15.5(9–30)	0.003	21 (15–30)	15 (9–30)	< 0.001

Abbreviations: FUBC: follow-up blood culture, ICU: intensive care unit, IQR: interquartile range, SOFA score: the sequential organ failure assessment score, SIRS: systemic inflammatory response syndrome, BSI: bloodstream infection, CR-GN: carbapenem-resistant gram-negative, MDR: multi-drug resistant, XDR: extensively drug-resistant, PDR: pan-drug resistant

* *Ochrobactrum anthropi, Achromobacter* spp., *Aeromonas* spp., *Delftia acidovorans, Moraxella catarrhalis, Raoultella* spp., *Pantoea* spp., *Shewanella* spp. ,*Sphingomonas* spp., *Ralstonia insidiosa, Burkholderia cepacia, Brucella* spp.

Patients were compared for mortality risk factors. SOFA score (OR:1.33; 95% CI, 1.23–1.44), CCI (OR:1.18; 95% CI, 1.08–1.28), hospital-acquired infections (OR:1.93; 95% CI, 1.08–3.46) and carbapenem-resistant GN-BSI (OR: 2.92; 95% CI, 1.72–4.96) were independent risk factors for mortality (Table 4).

**Table 4:** Mortality risk factors after propensity matching cohort

	Mortality n = 159	No Mortality n = 313	P value	Adjusted OR
Age (years), median (IQR)	67 (58–79)	63 (48.5)	0.002	Not included ^¶^
Male sex, n (%)	84 (52.8)	177 (56.5)	0.442	
Comorbidities, n (%)				
Diabetes mellitus	44 (27.7)	81 (25.9)	0.676	
Hypertension	74 (46.5)	116 (37.1)	0.047	Not included ^¶^
Chronic heart disease	47 (26.9)	63 (20.1)	0.022	Not included ^¶^
Cerebrovascular disease	21 (13.2)	39 (12.5)	0.818	
Chronic obstructive pulmonary disease	17(10.7)	33 (10.5)	0.960	
Malignancy	91 (57.2)	153 (48.9)	0.086	Not included ^¶^
Charlson comorbidity index score, median (IQR)	6 (4–8)	5 (2–6)	< 0.001	1.18 (1.08–1.28)
Clinical severity at index blood culture time, median (IQR)				
SOFA score	7 (4–9)	3 (1–5)	< 0.001	1.33 (1.23–1.44
SIRS	2 (1–3)	2 (1–3)	0.516	
ICU support, n (%)	121 (76.1)	106 (33.9)	< 0.001	Not included ^*^
Central venous catheter, n (%)	115(72.3)	154 (49.2)	< 0.001	0.79 (0.45–1.38)
Cardiac device, n (%)	10 (6.3)	10 (3.2)	0.115	
Prosthetic device, n (%)	6 (3.8)	14 (4.5)	0.722	
Site of BSI acquisition, n (%)				
Community-acquired	32 (20.1)	122 (39)	< 0.001	1.93 (1.08–3.46)
Nosocomial acquired	127 (79.9)	191 (61)		
Source of BSI, n (%)				
Primary BSI	66 (41.5)	134 (42.8)	0.786	
Secondary BSI	93 (58.5)	179 (57.2)		
CR-GN, n (%)	80 (55.9)	59 (20.4)	< 0.001	2.92 (1.72–4.96)
MDR, n (%)	41(25.8)	113 (36.1)	0.024	0.98 (0.58–1.65)
XDR, n (%)	46 (28.9)	30 (9.6)	< 0.001	Not included ^¶^
PDR, n (%)	5 (3.1)	4 (1.3)	0.161	
FUBC, n (%)	78 (49.1)	158 (50.5)	0.770	
FUBC positivity, n (%)	24 (30.8)	33 (20.9)	0.095	1.56 (0.69–3.53)
Persistent BSIs, n (%)	7 (9)	6 (3.8)	0.101	
Appropriate empirical antibiotic therapy, n (%)	76 (47.8)	170 (54.3)	0.181	

Nagelkerke R: 0.385, Hosmer-Lemeshow test: 0.608

^¶^ The variable covered by the other variables in the model was not included in the regression model. Age and comorbid diseases were not included in the model because they were parameters of CCIs. As all XDR-GNs were CR, XDR-GNs were not included in the model

* Variable with high correlation with other variables was not included in the regression model.ICU support was not included in the model due to its high correlation with the SOFA score (spearman, rho >0.75)

Abbreviations: ICU: intensive care unit, IQR: interquartile range, SOFA score: the sequential organ failure assessment score, SIRS: systemic inflammatory response syndrome, BSI: bloodstream infection, CR-GN: carbapenem-resistant gram-negative, MDR: multi-drug resistant, XDR: extensively drug-resistant, PDR: pan-drug resistant, FUBC: follow-up blood culture

In the FUBC group, the duration of effective antibiotic therapy (10 (4–16) vs. 15 (9–20), p < 0.001) and antibiotic consumption(DOT/1000 PD) for index BSIs (1090 (1000–1800) vs. 1375 (1000–2000), p = 0.002) was more than the non-FUBC group. Antibiotic consumption(DOT/1000 PD) within 1 month after index culture was also higher in the FUBC group (1609 (1000–2178) vs.2000 (1294–2769), p < 0.001). There was no relationship between FUBC result and duration of effective antibiotic therapy and total antibiotic consumption (p > 0.05) (Table 5).

**Table 5:** Comparison of antibiotic use according to FUBC results

	Duration of effective antibiotic therapy (days), median (IQR)	P value	DOT/1000 PD for antibiotics used in index BSIs median (IQR)	P value	DOT/1000 PD for antibiotics within 1 month after index culture	P value
No FUBC	10 (4–16)	< 0.001	1090 (1000–1800)	0.002	1609 (1000–2178)	< 0.001
FUBC	15 (9–20)		1375 (1000–2000)		2000 (1294–2769)	
Negative BSI	15 (10–20)	0.755	1351 (1000–1951)	0.098	2000 (1315–2647)	0.588
Positive BSI	14.5 (7–19)		1363 (983–2059)		2060 (1271–3112)	
Persistent BSI	15 (9–17)		1921 (1380–2786)		2033 (1503–3017)	

Abbreviations: DOT/1000 PD: days of therapy per 1000 patient days, FUBC: follow-up blood culture, BSI: bloodstream infection

The frequency of 30-day mortality was found to be 24.3% in 564 patients in the FUBCs group, and 36.3% in 248 patients without FUBC. 30-day mortality in the FUBC group was calculated with a type 1 error of 5% and a power of 91.6%.

## Discussion

In our study, although the prevalence of persistent BSI was 3.9% in GN-BSIs, FUBC was performed in approximately two-thirds of the patients. FUBC did not affect mortality. However, regardless of the FUBC result, FUBC was associated with a longer duration of antibiotic therapy and increased total antibiotic consumption.

The prevalence of persistent GN-BSI in the literature varies between 3% and 38% due to the difference in FUBC rates [3–6, 8–15]. The incidence of persistent infections increases in studies with low FUBC rates due to the high selection bias. Gienella et al. found the prevalence of persistent GN-BSI to be 38.5% with a 16% FUBC rate [8]. In contrast, the prevalence of persistent GN-BSI was only 3% in the study of Robinson et al., with a FUBC rate of 66% [14]. The high rate of FUBC in our study may reduce the possible risks of bias and enable a more accurate frequency of persistent GN-BSIs. Another risk of bias between studies of FUBCs is caused by differences in FUBC time. In previous studies, the time of FUBCs ranges from 24 h to 7 days [3–6, 8–15]. Delays in FUBCs may lead to changes in the frequency of persistence of GN-BSI due to the differentiation of treatment approaches (initiation of appropriate antibiotic therapy, duration of treatment, source control, etc.) for GN-BSI control. This risk of bias exists in our study as 2–7 days are used for FUBC. The prevalence of persistent GN-BSI has been evaluated independent of appropriate antibiotic therapy in published studies. In our study, the frequency of persistence decreased by half with appropriate antibiotic therapy. This decrease supports that FUBCs may not be necessary for persistent GN-BSI in patients receiving appropriate antibiotic therapy. On the other hand, FUBC can be recommended in patients with increased risk of persistent BSI; such as patients with high SOFA scores, patients with CVC, hospital-acquired infections, carbapenem-resistant and non-fermenter GN-BSIs and patients not receiving appropriate empirical therapy.

Different results have been reported in the literature for the association of FUBC with mortality in GN-BSI [2–15]. In two recently published meta-analyses, FUBC was associated with a lower risk of mortality. Low mortality risk may be associated with early detection of complications, early source control, and early initiation of appropriate antibiotic therapy in FUBC groups [16, 17]. However, in studies with low mortality risk in FUBC groups, the rates of FUBC (17–68%) were lower than in studies that did not affect mortality (67–89%) [3–13]. Longer duration of FUBC (up to 7 days) may lead to the misleading association of FUBC with a lower mortality risk due to early mortality before FUBC. To reduce this risk of bias, Gienella et al. matched the groups for SOFA score and FUBC times and found that FUBC was an independent variable for low risk of mortality. However, the researchers noted that a causal relationship between FUBC and mortality cannot be established due to the inability to completely rule out confounding risk factors for mortality and the lack of standard protocols for performing FUBC [8]. Another study, Mitaka et al. found no correlation between FUBC and mortality in patient groups matched for confounding factors. However, generalization of study results was not possible due to the insufficient power of the study [13]. In our study, in all cohorts, the FUBC group had less ICU support and lower SOFA scores. Patient groups were matched in SOFA score, similar to Giaenella et al., to reduce the effect of mortality on FUBC. When mortality risk factors were evaluated in a more homogeneous subgroup, no relationship was found between FUBC and mortality.

In previous studies, antibiotic treatment duration is 2–5 days longer in FUBC groups because of waiting for FUBC results or starting new antibiotics according to the result [16]. However, the comparison of treatment durations in studies is based on the first effective antibiotic, and the effect of sequential treatments is often ignored [4–6, 13, 15]. FUBC positivity, some of which may be contaminated, reported between 4% and 49% in studies may affect antibiotic consumption [6–15]. In our study, besides the effective treatment duration, the total antibiotic consumption within 30 days after index blood culture was compared via DOT/1000 PD. FUBC was associated with long-effective antibiotic duration and increased total antibiotic consumption. However, unequal follow-up durations between FUBC groups due to death or discharge complicated the causal relationship between FUBC and antibiotic consumption. There was no correlation between FUBC results and total antibiotic consumption. In our opinion, this situation is related to the lack of diagnostic and antibiotic stewardship algorithms in our center. The FUBC decision is based on individual clinical decisions, monitoring and sharing of FUBC results cannot be provided adequately, especially in non-critical patients. This result supported that routine FUBC in GN-BSIs is not recommended without a diagnostic and antimicrobial stewardship program due to the limited effect on antibiotic treatment.

One of the strengths of our study, contrary to the other studies in the literature, is the evaluation of the frequency of persistent BSI after appropriate antibiotic therapy. The lower frequency of persistent GN-BSI in patients receiving appropriate antibiotic therapy should be considered in the development of the standard FUBC recommendation for GN-BSI. Comparing the relationship between FUBC and mortality in a more homogeneous patient group and evaluating total antibiotic consumption, including sequential antibiotic treatments, are the other strengths of our study. Especially due to high multidrug resistance in GNs, the effect of FUBC antibiotic consumption should be analyzed in more detail and should be considered within the scope of FUBC antimicrobial stewardship programs. However, our study has limitations. First, due to the retrospective nature of our study, not all of the confounding factors that could have affected performing FUBC could be evaluated. Factors arising from the behavior of clinicians cannot be ignored, especially due to the lack of a standard approach for FUBC and the uncertain clinical indications for FUBC. Second, our study has sufficient power to show the difference in mortality in all patient populations. However, it is underpowered to show the difference in mortality of approximately 1% in subgroups obtained after matching. Third, the effect of FUBC on antibiotic consumption was assessed within 30 days after the index culture. Since other infections in this period could not be completely excluded due to the retrospective design of the study, a causal relationship between FUBC and antibiotic consumption could not be established.

In conclusion, routine FUBC should not be recommended because of the low prevalence of persistent infections in patients under appropriate antibiotic therapy and the lack of relationship between FUBC and mortality. Regardless of its results, FUBC should not be routinely used without antibiotic stewardship programs because of its association with high antibiotic consumption.

### Future directions

Our study supported that FUBC increased antibiotic use in GN-BSI despite its limited effect on clinical outcomes. The uncertainty of clinical indications of FUBC in GN-BSIs suggests that reflex responses to FUBC results may lead to inappropriate antibiotic use. In our opinion, future studies should be conducted to define the FUBC indications. The 7.9% persistent and 14% positive FUBC results in our study suggest that future studies can be conducted to identify patients who may benefit from FUBC. Evaluation of the relationship between FUBC-positive results (selection for antimicrobial resistance or breakthrough infection) and antibiotics used for index BSI based on antibiotic-related collateral damage in planned studies will be useful in determining FUBC indications as well as clinical criteria.

## Data Availability

The data that support the findings of this study are available on request from the corresponding author. Restrictions apply to the availability of these data, which were used under license for the current study, and thus are not publicly available. Data are, however, available from the authors upon reasonable request and with permission from the Gazi University Faculty of Medicine Clinical Research and Ethics Committee.
